# Functional immune profiling translates T cell dynamics into predictive biomarkers for myeloma immunotherapy

**DOI:** 10.1038/s41375-026-03047-5

**Published:** 2026-07-16

**Authors:** Frederik Herzberg, Feiteng Lu, Michael Korenkov, Vincent Fregona, Carin Didoss, Nina Heisterkamp, Maximilian Sprechert, Alexandra Polici, Clarissa Heck, Simon Haas, Jan Krönke, Ulrich Keller, Stephan R. Bohl

**Affiliations:** 1 Department of Hematology, Oncology and Cancer Immunology, Charité – Universitätsmedizin Berlin, Corporate Member of Freie Universität Berlin and Humboldt-Universität Berlin, Berlin, Germany; 2 Max-Delbrück-Center for Molecular Medicine, Berlin, Germany; 3 Berlin Institute of Health (BIH) at Charité – Universitätsmedizin Berlin, Berlin, Germany; 4 German Cancer Consortium (DKTK), Partner Site Berlin, Germany and German Cancer Research Center (DKFZ), Heidelberg, Germany; 5 Precision Healthcare University Research Institute, Queen Mary University of London, London, UK; 6 Department of Hematology, Oncology, University Medicine Greifswald, Stem Cell Transplantation and Palliative Care, Greifswald, Germany; 7 Cluster of Excellence ImmunoPreCept, Charité – Universitätsmedizin Berlin, Berlin, Germany

**Keywords:** Targeted therapies, Biotechnology, Immunotherapy

## Abstract

Bispecific T cell engagers (TCE) targeting BCMA or GPRC5D have improved outcomes in relapsed/refractory multiple myeloma (MM), yet up to 40% of patients are primarily refractory, and predictive biomarkers are scarce. To address this unmet clinical need, we developed an image-based, high-content ex vivo assay using patient-derived bone marrow mononuclear cells exposed to teclistamab or talquetamab. Multiplex immunofluorescence enabled single-cell quantification of plasma cell lysis, T cell expansion, T cell morphology, and spatial T cell–plasma cell engagement. Across all ex vivo*-*treated patient samples, we identified three functional response phenotypes: non-responder, cytotoxic, and cytotoxic-expansive. Based on this classification, we delineated a morphologic T cell activation trajectory from resting to polarized and effector states. Progression along this trajectory reflected ex vivo plasma cell killing efficacy and distinguished productive from abortive immunological synapses. Importantly, ex vivo response phenotypes stratified clinical response and time on therapy in patients receiving TCE monotherapy, positioning image-based ex vivo profiling as a functional biomarker for immune competence and therapy stratification in MM.

**Figure:**
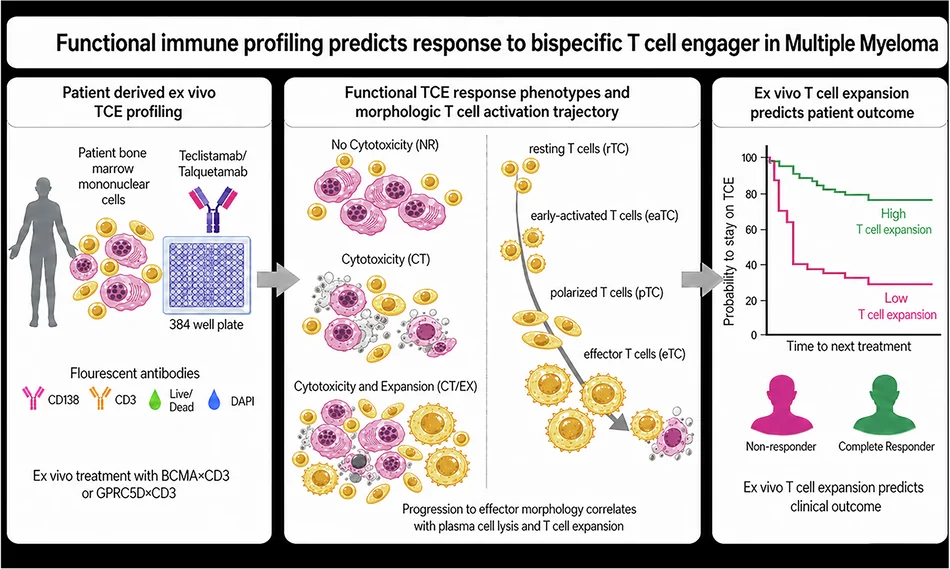

## Introduction

Multiple myeloma (MM) is a malignancy of clonally proliferating plasma cells that accumulate in the bone marrow [1]. It is the second most common hematologic cancer in Western countries and remains incurable [1, 2]. Most patients ultimately die of relapsed or refractory disease despite multiple lines of therapy [1]. Bispecific T cell engagers (TCE) targeting B cell maturation antigen (BCMA) or G-protein coupled receptor family C group member D (GPRC5D) have reshaped treatment for relapsed or refractory MM, inducing deep and durable remissions in a subset of patients [3–5]. By crosslinking CD3 on T cells to myeloma surface antigens, TCEs promote immune synapse formation, T cell activation and clonal T cell expansion [6, 7]. Yet, clinical trials as well as real-world data suggest that up to 40% of patients are primary refractory to TCE therapy, and many initial responders relapse within months due to secondary resistance [8–11].

Mechanisms of secondary resistance, such as target antigen loss or downregulation and T cell exhaustion, are increasingly well documented [12–16]. In contrast, the cellular determinants of primary resistance remain incompletely understood. Clinical features such as disease burden, extramedullary disease, or prior immunotherapy exposure associate with worse outcomes but do not fully explain inter-patient variability in TCE efficacy [9, 11, 15, 17]. Recent single-cell transcriptomic studies have highlighted the importance of naïve-to-effector T cell activation programs and MHC-I-dependent synapse formation for TCE responsiveness, whereas early treatment failure was linked with the enrichment of terminally exhausted T cells in the bone marrow [18]. While transcriptomic profiling offers mechanistic insights, these approaches provide only static molecular snapshots and lack the spatial or temporal resolution necessary to capture dynamic processes like synapse formation or cytolytic engagement [19]. Moreover, their high cost and complexity limit their utility as real-time biomarkers for clinical decision-making [19].

Functional ex vivo drug profiling offers a powerful complement to molecular profiling, enabling real-time assessment of treatment susceptibility in patient-derived samples [20–23]. In hematologic malignancies, image-based approaches have shown predictive value for multiple drug classes, and studies like the EXALT-I trial have demonstrated that such approaches can scale to clinically useful throughput [20, 21, 24]. High-content imaging allows scalable, unbiased single-cell readouts of cancer and immune cell behavior, integrating population-level shifts (tumor cell lysis, T cell expansion) with morphologic features (cytoskeletal remodeling, nuclear enlargement, cell polarization) and cell-cell interactions in situ, attributes linked to T cell activation and killing efficacy [25–31]. Despite these preliminary data, a high-content imaging assay has not been systematically applied to primary MM bone marrow samples to delineate TCE response phenotypes, nor integrated with spatial metrics of T cell–plasma cell engagement and clinical outcomes.

Here, we developed an image-based, high-content ex vivo assay to functionally profile responses to Teclistamab (TEC, BCMA×CD3) and Talquetamab (TAL, GPRC5D×CD3) in MM patient-derived bone marrow mononuclear cells (BMMCs). This ex vivo assay translates TCE pharmacodynamics into quantitative single-cell readouts, revealing TCE response phenotypes, a T cell activation trajectory, and biomarkers that connect ex vivo response with patient clinical outcome.

## Methods

### Patient data, clinical response assessment and ethical approval

Bone marrow aspirates were collected from 40 patients (Table 1) from March 2024 to March 2026. 12 patient samples were collected before TCE monotherapy. Response to TCE was assessed using the International Myeloma Working Group uniform response criteria. Data cut for time to next treatment was 31st December 2025. The study was approved by the Ethics Committee of Charité—Universitätsmedizin Berlin (approval number EA1/152/10). Written informed consent was obtained from all participants in accordance with the Declaration of Helsinki. Clinical annotations were retrieved from the hospital information system.

**Table 1:** Patient characteristics of all samples treated ex vivo (dx=new diagnosis, r/r=relapsed/refractory, EM= extra medullary disease; IMID= immunomodulatory drug PI= proteasome inhibitor).

ID	Age (years)	Disease Stage	ISS	EM	Cytogenetics	IMiD	PI	CD38mAB	Drug Class Refractory
1	56	dx	1	no	standard	n.a.	n.a.	n.a.	n.a.
2	72	dx	2	no	standard	n.a.	n.a.	n.a.	n.a.
3	58	dx	1	no	standard	n.a.	n.a.	n.a.	n.a.
4	55	dx	2	no	highrisk	n.a.	n.a.	n.a.	n.a.
5	76	dx	2	no	standard	n.a.	n.a.	n.a.	n.a.
6	76	dx	2	no	standard	n.a.	n.a.	n.a.	n.a.
7	42	dx	1	no	standard	n.a.	n.a.	n.a.	n.a.
8	55	dx	2	no	standard	n.a.	n.a.	n.a.	n.a.
9	56	dx	2	no	standard	n.a.	n.a.	n.a.	n.a.
10	56	dx	1	no	highrisk	n.a.	n.a.	n.a.	n.a.
11	80	dx	2	yes	unknown	n.a.	n.a.	n.a.	n.a.
12	62	r/r	1	no	highrisk	refractory	exposed	refractory	2
13	63	r/r	3	yes	unknown	refractory	refractory	refractory	3
14	73	r/r	3	no	highrisk	refractory	refractory	refractory	5
15	61	r/r	1	yes	standard	refractory	exposed	refractory	2
16	64	r/r	2	no	standard	refractory	refractory	refractory	5
17	72	r/r	2	no	unknown	refractory	exposed	refractory	2
18	74	r/r	3	no	unknown	refractory	refractory	refractory	3
19	75	r/r	3	yes	standard	refractory	refractory	refractory	3
20	61	r/r	2	no	standard	refractory	refractory	refractory	5
21	58	r/r	1	no	standard	refractory	refractory	refractory	3
22	70	r/r	3	yes	highrisk	refractory	refractory	refractory	5
23	71	r/r	3	yes	standard	refractory	refractory	refractory	3
24	64	r/r	2	no	standard	refractory	refractory	refractory	3
25	80	r/r	2	no	unknown	refractory	refractory	refractory	3
26	64	r/r	1	no	standard	refractory	refractory	refractory	5
27	61	r/r	2	no	highrisk	refractory	refractory	refractory	3
28	59	r/r	1	no	standard	refractory	exposed	exposed	1
29	64	r/r	2	no	standard	refractory	refractory	refractory	5
30	54	r/r	3	no	standard	refractory	exposed	exposed	1
31	65	r/r	2	no	highrisk	refractory	refractory	refractory	3
32	52	r/r	2	no	standard	refractory	refractory	refractory	3
33	78	r/r	3	no	unknown	refractory	refractory	refractory	3
34	69	r/r	3	no	unknwon	refractory	refractory	refractory	3
35	67	r/r	2	no	standard	refractory	refractory	refractory	5
36	78	dx	2	no	standard	n.a.	n.a.	n.a.	n.a.
37	56	dx	2	no	standard	n.a.	n.a.	n.a.	n.a.
38	52	dx	2	no	standard	n.a.	n.a.	n.a.	n.a.
39	52	dx	2	no	standard	n.a.	n.a.	n.a.	n.a.
40	82	r/r	3	no	highrisk	refractory	refractory	refractory	5

### Sample processing and cell isolation

Bone marrow aspirates were collected in EDTA tubes and processed within 24 h. Mononuclear cells (BMMCs and PBMCs) were isolated using Ficoll-Paque density gradient (Sigma-Aldrich, St. Louis, MO, USA) centrifugation, followed by red blood cell lysis (Sigma-Aldrich, St. Louis, MO, USA). Isolated cells were washed, counted, and used immediately or within 24 h.

### Cell culture

MM1.S and OPM2 cell lines, BMMCs and peripheral blood mononuclear cells (PBMCs) were cultured in RPMI 1640 with GlutaMax (Thermo Fisher Scientific, Waltham, MA, USA) media supplemented with 10% fetal bovine serum and 1% penicillin-streptomycin at 37 °C and 5% CO_2_. Cell lines were regularly authenticated and tested for mycoplasma.

### Ex vivo drug treatment of BMMC and PBMC-cancer-cell cocultures

Patient-grade TEC and TAL (Janssen, Beerse, Belgium), purchased from the clinical pharmacy, were tested at four increasing concentrations in technical triplicates using 384-well plates. TEC was tested at 70, 7, 0.7 and 0.07 nM, TAL was tested at 270, 27, 2.7, 0.27 nM, or in combination. BMMCs (10⁶/mL) or PBMC-myeloma co-cultures (1:10 ratio) were seeded in 384-well PhenoPlates (Revvity, Waltham, MA, USA) and treated for 24, 72, or 120 h w/o soluble BCMA (Sigma Aldrich, SRP3010-20UG). DPBS served as vehicle control. Plates were sealed with Breathe-Easy membranes (Sigma-Aldrich, St. Louis, MO, USA) to prevent evaporation.

### Immunostaining and imaging

After incubation, cells were washed and stained with LIVE/DEAD Fixable Dye (Thermo Fisher Scientific, Waltham, MA, USA), fixed with Cytofix, permeabilized with Cytoperm (Becton Dickinson, Franklin Lakes, NJ, USA), and stained with CD3-PE (clone SK7, BioLegend, San Diego, CA, USA), CD138-APC (clone B-A38, Beckman Coulter, Brea, CA, USA) and DAPI (Sigma-Aldrich, St. Louis, MO, USA). Cells were stained in the dark overnight at 4 °C. The next day, 25 non-overlapping fields per well were imaged at 20× magnification using an Operetta CLS High-Content Analysis System (Revvity, Waltham, MA, USA). DAPI (435-480 nm), EGFP (500-550 nm), PE (570-630 nm) and APC (650-760 nm) channels were acquired with constant LED exposure and power settings.

### Image analysis

Image analysis was performed with Harmony 5.1 software (Revvity). First, all images were flat-field corrected. Cells were detected by DAPI nuclear stain (Find nuclei function). To detect different markers, a cell area was defined from the nuclear mask. By linear intensity thresholding of APC, PE and GFP intensity values, cells were assigned to viable, CD138⁺ and CD3⁺ cell populations. For interacting CD3⁺ cells, the minimum distances between the membranes of CD3⁺ and CD138⁺ cells were calculated. A CD3⁺ cell that was within 1 µm of a CD138⁺ cell was classified as interacting CD3⁺. For single-cell morphologic profiling, Cell Painting Properties were calculated from DAPI and PE channels using the Harmony Cell Painting module [30, 32]. Following image analysis, all properties were exported to perform downstream analysis. For synergy calculation and illustration https://synergyfinder.org/ was used.

### Single-cell morphology analysis

For single-cell morphology analysis, exported Cell Painting features from all samples, compounds and concentrations were loaded into RStudio (Posit PBC; R version 4.4.2). For each sample and condition, 250 cells were randomly sampled into the analysis data frame. Next, following prior reports [33], the data frame was filtered for high-quality cells, removing all cells missing 30 or more features and poorly segmented cells with a “Cell Region Area” smaller than 30 or greater than 250 and a “Cell Region Roundness” below 0.6. Next, highly correlated features (r > 0.9) were removed from the data frame to decrease feature redundancy. Remaining features were z-scaled and subjected to principal component analysis. The top 35 principal components were used for hierarchical clustering and Uniform Manifold Approximation and Projection (UMAP).

### Calculation of cell fractions, interaction score, drug response score and iAUC_norm_

Cell and subpopulation fractions were calculated per well and normalized to control means. T cell–plasma cell engagement was quantified as log₂ enrichment of observed versus expected interactions, with expected values derived from the harmonic mean of CD3⁺ and CD138⁺ cell frequencies [34]. Enrichment values were baseline-corrected and averaged across replicates. Drug response scores (DRS) were defined as 1 minus the geometric mean of control-normalized fractions across concentrations (positive = reduction, negative = expansion). Morphologic cluster fractions and interacting CD3⁺ T cells were summarized by baseline-corrected, control-centered area under the log₁₀-dose curve (iAUCnorm).

### Statistics

Sample size was not predetermined by formal statistical methods but was guided by patient sample availability and aligned with previous ex vivo imaging studies [16, 25]. Samples were included if the MM diagnosis was confirmed, at least two time points were measured, and quality criteria were met (viability >50%, >5% CD138⁺ cells). Data visualization and statistical testing were performed using GraphPad Prism 10 (GraphPad Software, Inc) and RStudio with standard R packages. For comparisons between two independent groups, the Mann–Whitney U test was used. Paired data were analyzed using paired t-tests or Wilcoxon matched-pairs signed-rank tests, as appropriate. Comparisons across more than two groups were performed using the Kruskal–Wallis test. Response curves were compared using two-way ANOVA. Correlations were assessed using Spearman’s rank correlation. Spearman’s r is reported with 95% confidence intervals. Survival analyses were performed using the log-rank test and Cox proportional hazards models. Data are displayed with mean ± standard deviation if not indicated otherwise by the figure legend. Results were considered significant if *p* < 0.05.

## Results

### An image-based, high-content ex vivo assay delineates functional TCE response phenotypes

**Fig. 1:**
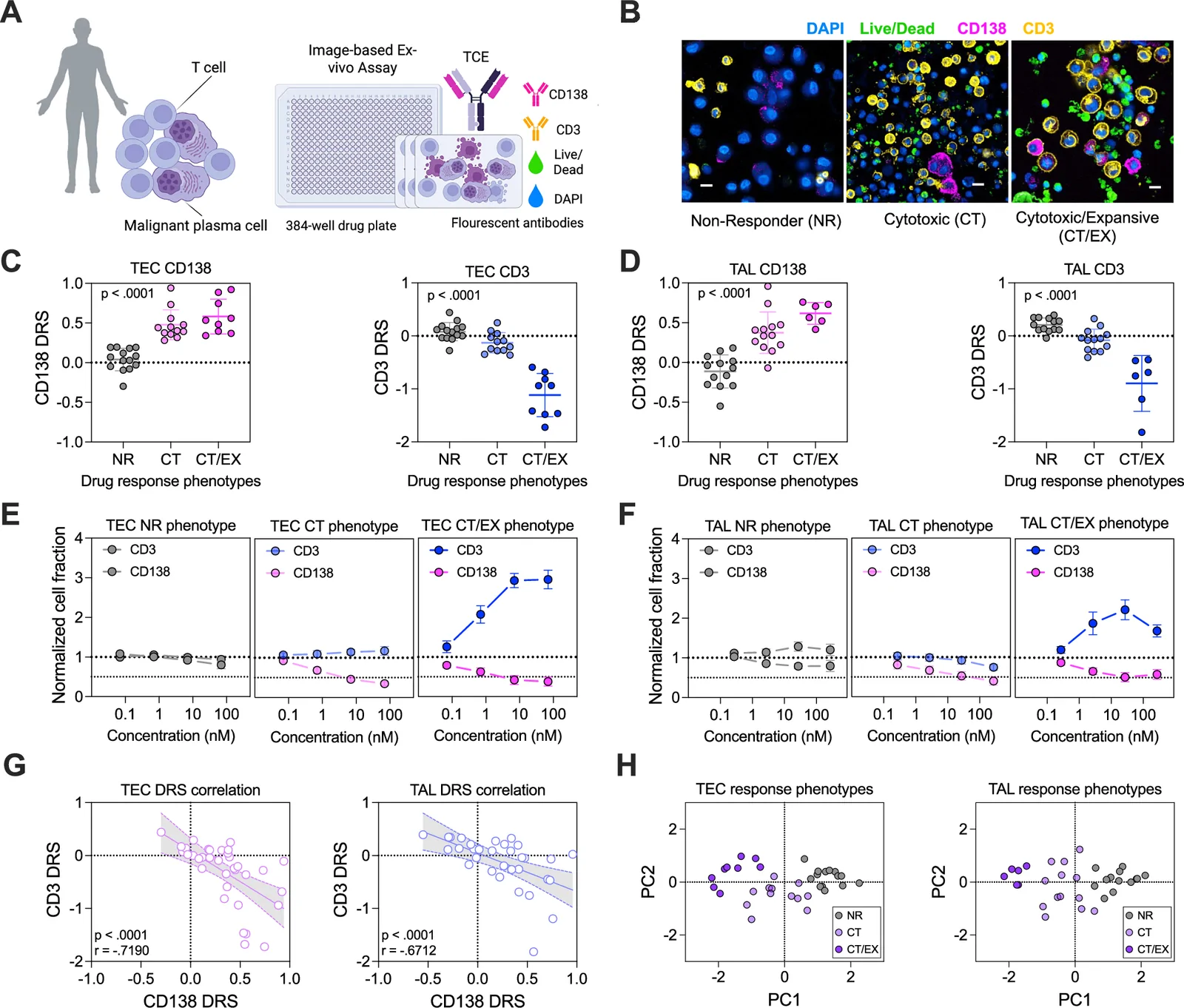
A high-content imaging assay reveals distinct functional TCE response phenotypes. **A** Overview of the image-based ex vivo drug-screening workflow using MM BMMCs. **B** Representative images of patient BMMCs after 120 h TEC treatment (70 nM) showing the three ex vivo response phenotypes. CD138⁺ plasma cells (magenta), CD3⁺ T cells (yellow), nuclei (DAPI, blue), and dead cells (LIVE/DEAD, green) are shown. Linear contrast settings were applied identically across images. Scale bar, 10 µm. **C, D** CD138-DRS and CD3-DRS by response phenotype for TEC (C; *n* = 36 patient samples) and TAL (**D**; *n* = 33 patient samples) at 120 h. Dose–response curves of control-normalized CD3⁺ and CD138⁺ fractions by response phenotype for TEC (**E**) and TAL (**F**). **G** Correlation of CD3-DRS and CD138-DRS for TEC (left; *n* = 36) and TAL (right; *n* = 33). **H** Principal component analysis of clustered TEC-treated (left) and TAL-treated (right) samples; each point represents one patient sample. Data are shown as mean ± SD (**C, D**) and mean ± SEM (**E, F**). Lines indicate best fit with 95% confidence intervals (**G**). Statistical analyses: Kruskal–Wallis test with multiple-comparison correction (**C, D**), Spearman’s rank correlation (**G**). Schematic representation in A created with BioRender.com. Abbreviations: MM multiple myeloma, BMMCs bone marrow mononuclear cells, DRS drug response score, TEC teclistamab, TAL talquetamab.

We established an image-based, high-content ex vivo assay to profile TCE responses in primary MM BMMCs. Cells acquired from patients at various disease stages (Table 1) were treated with four increasing concentrations of TEC and TAL for up to 120 h. Multiplex immunofluorescence imaging (CD138, CD3, viability dye, and DAPI) enabled quantification of viable CD138⁺ plasma cells and CD3⁺ T cells (Fig. 1A**)**.

TCE exposure induced progressive CD138⁺ plasma cell lysis and CD3⁺ T cell expansion, most pronounced at 120 h (Supplementary Fig. 1B-C). Based on these metrics, we selected 120 h as the primary assay endpoint.

To quantify response magnitude, we calculated DRS for viable CD138⁺ plasma cells and CD3⁺ T cells. The DRS metric summarizes treatment effects across concentrations by calculating the geometric mean of control-normalized relative cell fractions [25]. A CD138 DRS > 0 indicates cytotoxicity, whereas CD3 DRS < 0 indicates proliferative expansion. After scaling, centering and clustering DRS values from *n* = 35 TEC-treated and *n* = 33 TAL-treated patient samples, we identified three ex vivo drug response phenotypes, which we termed non-responder (NR), cytotoxic (CT) and cytotoxic-expansive (CT/EX) (Fig. 1B). NR samples showed neither plasma cell lysis nor T cell expansion. In contrast, CT/EX samples demonstrated marked plasma cell lysis and robust T cell expansion. CT samples exhibited significant plasma cell lysis without accompanying T cell expansion (Fig. 1C–F). Consistent with these response phenotypes, CD138 DRS inversely correlated with CD3 DRS (Fig. 1G), indicating that greater plasma cell lysis (positive CD138 DRS) was associated with greater T cell expansion (negative CD3 DRS). Together, these data reveal substantial heterogeneity in functional ex vivo TCE responses, spanning a continuum from NR to CT and CT/EX phenotypes (Fig. 1H). There was no difference seen between response phenotypes regarding plasma cell percentage (Supplementary Fig. 1D–F). Further on, based on recent clinical findings [35], the combinations of Tec/Tal provide efficient cell killing synergism ex vivo (Supplementary Fig. 1G).

### Morphologic profiling resolves a resting-to-effector trajectory of T cell activation correlating with TCE response ex vivo

**Fig. 2:**
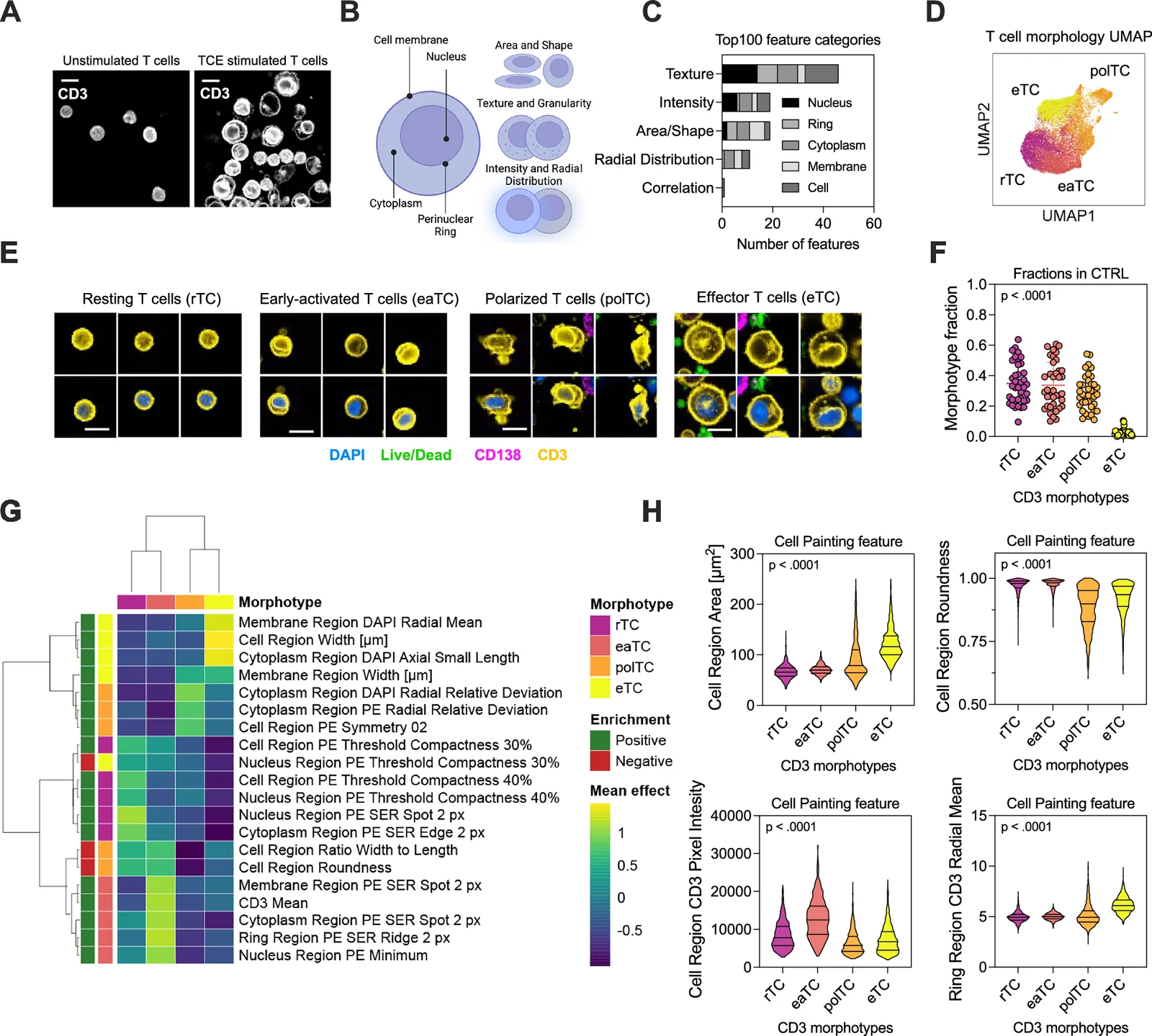
Morphologic profiling reveals a continuum of T cell activation states. **A** Representative images of CD3⁺ T cells in control and TCE-treated conditions at 120 h. The CD3 signal is shown in white. Scale bar, 10 µm. **B** Schematic of the cellular compartments quantified by the Cell Painting module. **C** Categories and distribution of the top 100 morphology features used for clustering. **D** UMAP projection of single-cell morphology features colored by T cell morphotype; *n* = 44,675 cells. **E** Representative images of the four TCM: rTC, eaTC, polTC, and eTC. CD3⁺ T cells are shown in yellow. Linear contrast settings were applied identically across images. Scale bar, 10 µm. **F** Morphotype fractions in control wells at 120 h; *n* = 36 patient samples. **G** Heatmap showing the five most variable features across TCM. **H** Selected defining features across TCM (Cell Region Area [µm²], Cell Region Roundness, Cell Region CD3 Pixel Intensity, Ring Region CD3 Radial Mean); *n* = 1000 randomly sampled cells per morphotype. Data are presented as mean ± SD in (**F**), as mean effect in (**G**) and as violin plots in (**H**). Statistical analyses: Kruskal–Wallis test with multiple-comparison correction (**F–H**). Schematic representation in B created with BioRender.com. Abbreviations: TCM T cell morphotype, rTC resting T cell, eaTC early activated T cell, polTC polarized T cell, eTC effector T cell.

To examine whether differences in T cell activation underlie this heterogeneity, we systematically profiled CD3⁺ T cell morphology across untreated and TCE-treated conditions. Using CD3 and DAPI channels from our high-content imaging dataset (TEC, *n* = 35; TAL, *n* = 32; multiple concentrations), we implemented a modified Cell Painting pipeline to extract 454 single-cell morphology features (Fig. 2A and Supplementary Fig. 2A) [30, 32]. These features (area/shape, signal intensity, radial distribution and texture) were captured in nuclear, perinuclear, cytoplasmic, and membrane compartments (Fig. 2B, C and Supplementary Fig. 2B). Fixation and permeabilization allowed detection of both surface and cytoplasmic CD3, enabling visualization of T cell receptor (TCR)/CD3 localization dynamics [36–40].

Unsupervised clustering identified four T cell morphotypes (TCM): resting, early-activated, polarized and effector (Fig. 2D–F). In line with prior reports on ex vivo T cell morphology [26, 27, 41], we annotated TCM based on their defining morphologic features and function (Fig. 2G). Resting T cells appeared small and spherical with compact nuclei, dense cytoplasm, and surface restricted CD3. Early-activated T cells were similar in size but less compact, with the highest overall CD3 intensity and redistribution from a uniform membrane pattern to heterogeneous signals across membrane, cytoplasm, and perinuclear regions. Polarized T cells were elongated and asymmetric, with broadened membrane regions and CD3 redistributed to their cytoplasm and perinuclear zone. Effector T cells were the least compact and most expanded, featuring enlarged nuclei, dense cytoplasmic CD3 aggregates, and a bright perinuclear CD3 ring (Fig. 2E–H).

**Fig. 3:**
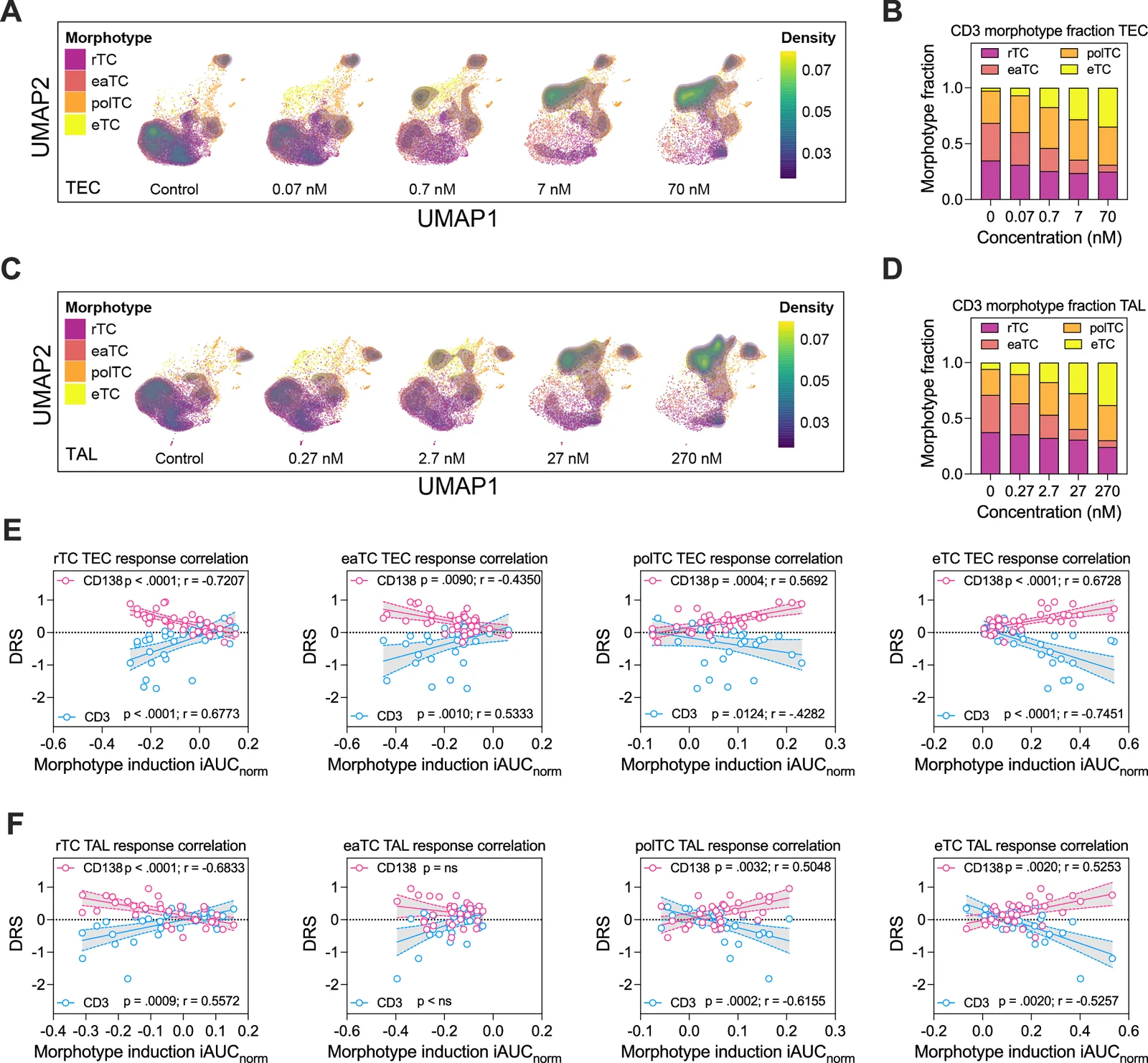
Morphologic T cell activation correlates with TCE response. UMAP density overlays showing dose-dependent shifts in TCM induced by TEC (**A**; 120 h; *n* = 44,675 cells) and TAL (**C**; 120 h; *n* = 40,463 cells). **B, D** Quantification of concentration-dependent changes in TCM fractions for TEC (**B**; *n* = 36 patient samples) and TAL (**D**; *n* = 33 patient samples). Correlations between TCM iAUCnorm values and CD3-DRS/CD138-DRS for TEC (**E**; *n* = 36) and TAL (**F**; *n* = 33) across rTC, eaTC, polTC, and eTC. Data are presented as a mean stacked bar plot in (**B, D**). Line of best fit presented as mean + 95% CI in (**E, F**). Statistical analyses: Spearman’s rank correlation (**E, F**). Abbreviations: TCM T cell morphotype, rTC resting T cell, eaTC early activated T cell, polTC polarized T cell, eTC effector T cell, iAUCnorm, baseline-corrected control-centered area under the log₁₀-dose curve, TEC teclistamab, TAL talquetamab.

Based on their feature distribution, these four TCM formed a morphologic continuum of T cell activation, ranging from resting to effector states. Consistent with this continuum, exposure to both TEC (Fig. 3A, B) and TAL (Fig. 3C, D) drove systematic, dose-dependent shifts in TCM distribution. Early-activated T cells decreased, whereas effector T cells increased across all samples. Polarized T cells increased modestly and became more heterogeneous, whereas resting T cells contracted into a small residual cluster at higher doses.

To assess whether these morphologic shifts are associated with ex vivo TCE activity, we computed a control-centered iAUC_norm_ for each TCM (Fig. 3E, F). Depletion of resting T cells and induction of polarized and effector TCM correlated strongly with ex vivo TCE response, i.e., greater plasma cell lysis and T cell expansion. Conversely, samples maintaining high fractions of resting T cells with limited induction of polarized or effector T cells exhibited minimal T cell expansion and plasma cell lysis. While changes in resting, polarized and effector TCM were strongly associated with ex vivo TCE sensitivity, early-activated T cells declined in all samples regardless of TCE response. Stratification by ex vivo response phenotype confirmed this observation: Polarized and effector TCM increased significantly in CT/EX phenotype samples while resting TCM declined compared to NR phenotype samples (Supplementary Fig. 3A, B). Together, these patterns delineate a TCE-induced morphologic activation trajectory, in which progression from resting to effector states reflected ex vivo TCE responses.

### Synaptic engagement metrics distinguish productive from abortive responses

**Fig. 4:**
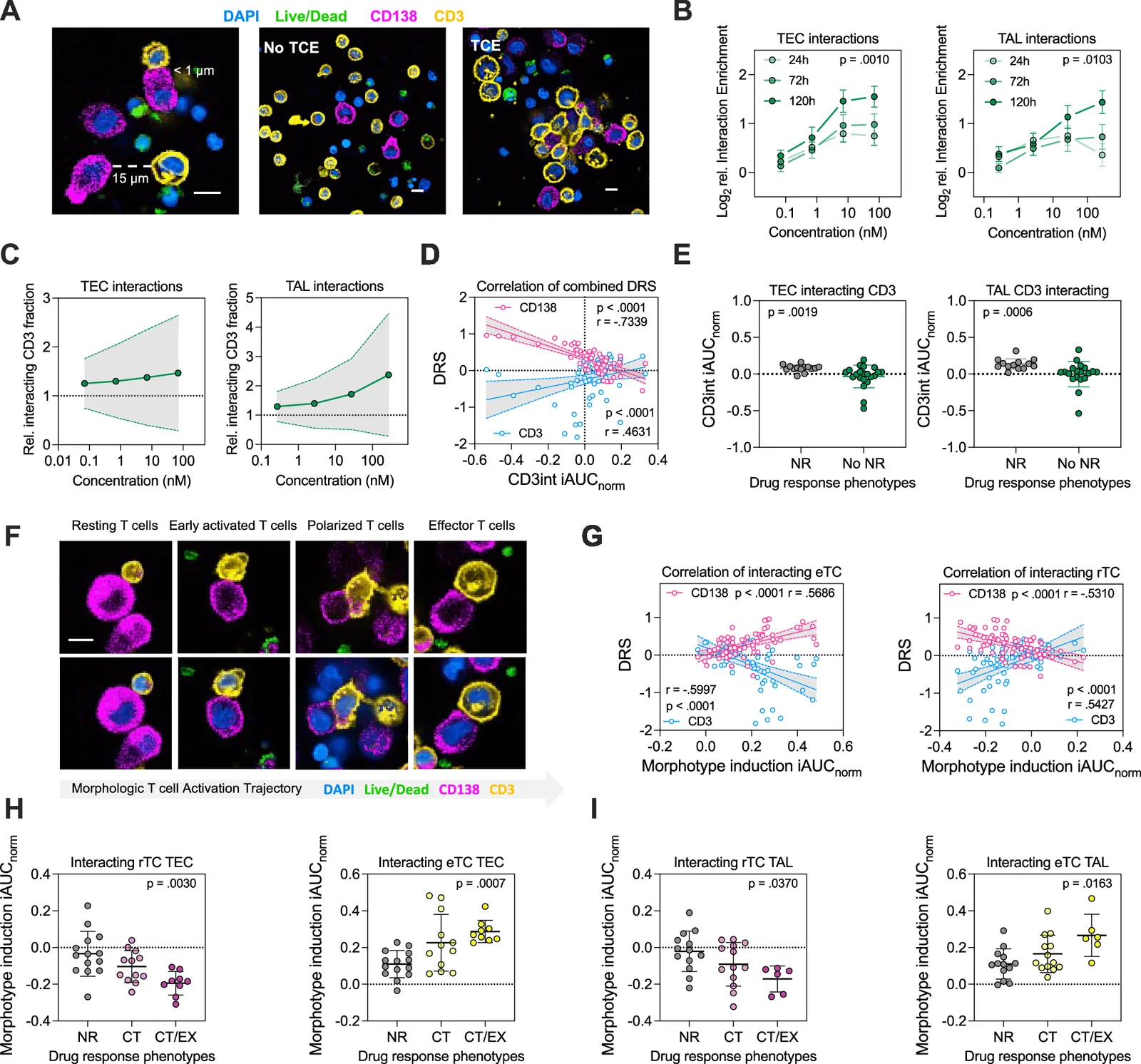
Functional synapse dynamics distinguish responding from non-responding samples. **A** Representative images illustrating CD3⁺–CD138⁺ interactions at 120 h. A CD3⁺ T cell was classified as interacting if the minimum membrane-to-membrane distance to a CD138⁺ plasma cell was ≤1 µm. CD138⁺ plasma cells (magenta) and CD3⁺ T cells (yellow) are shown. Linear contrast settings were applied identically across images. Scale bar, 10 µm. **B** Interaction enrichment (log₂ observed/expected) over time (24, 72, 120 h) for TEC (left) and TAL (right); *n* = 10 patient samples measured longitudinally. **C** Dose–response curves of the control-normalized fraction of interacting CD3⁺ T cells at 120 h for TEC (*n* = 36) and TAL (*n* = 33). **D** Correlations of CD3-DRS and CD138-DRS with CD3int iAUCnorm for TEC and TAL; *n* = 69 total sample–drug measurements. **E** CD3int iAUCnorm by response phenotype for TEC (*n* = 36) and TAL (*n* = 33). **F** Representative images of interacting TCM. Scale bar, 10 µm. **G** Correlations of iAUCnorm values for interacting rTC and eTC with CD3-DRS/CD138-DRS for TEC (*n* = 36) and TAL (*n* = 33). Interacting rTC and eTC iAUCnorm by response phenotype for TEC (**H**; *n* = 36) and TAL (**I**; *n* = 33). Data are presented as mean ± SD in (**C, E, H, I**) and as mean ± SEM in (**B, F**). Line of best fit presented as mean + 95% CI in (**D, G**). Statistical analyses: two-way ANOVA with Tukey’s multiple-comparison test (**B**), Mann–Whitney U test (**E**), Spearman’s rank correlation (**D, G**), Kruskal–Wallis test (**H, I**). Abbreviations: CD3int interacting CD3⁺ fraction, CT cytotoxic, CT/EX cytotoxic-expansive, NR non-responder, TEC teclistamab, TAL talquetamab, TCM T cell morphotype, rTC resting T cell, eTC effector T cell.

Morphologic T cell analysis suggested that the extent of progression toward the effector state was closely associated with T cell expansion and plasma cell lysis. However, morphology alone may not capture whether T cells form functional contacts with their targets. Therefore, we quantified spatial synapse engagement by labeling a CD3⁺ cell as interacting when its membrane lay within ≤1 µm of a CD138⁺ membrane (Fig. 4A) [25, 42].

To account for treatment-driven shifts in cell composition, we calculated a log_2_ interaction enrichment score by adjusting observed T cell–plasma cell interactions for expected cell abundances and compared it to the normalized fraction of interacting CD3⁺ T cells within the T cell pool [34]. Across longitudinally measured samples, mean interaction enrichment increased from 24 to 120 h (Fig. 4B), whereas the normalized uncorrected fraction of interacting CD3⁺ T cells decreased over the same interval (Supplementary Fig. 4A). At 120 h, the normalized uncorrected fraction of interacting CD3⁺ T cells exhibited increasing dose-dependent variance across samples; a pattern not observed for the interaction enrichment (Fig. 4C and Supplementary Fig. 4B). Correlating changes in the interacting CD3⁺ fraction (CD3int iAUC_norm_) and interaction enrichment with plasma cell lysis and T cell expansion revealed that a decrease in interacting T cells within the T cell compartment was strongly correlated with increased plasma cell lysis and T cell expansion at 120 h (Fig. 4D, and Supplementary Fig. 4C). Consistently, ex vivo responding samples (CT and CT/EX) showed significantly lower CD3int iAUC_norm_ values in direct comparison with NR phenotype samples (Fig. 4E).

To further characterize synaptic engagement, we integrated spatial interaction metrics with morphologic profiling (Fig. 4F, G). TCE exposure shifted the morphologic profiles of interacting T cells along the morphologic activation trajectory (Fig. 4H). Among interacting T cells, responding samples displayed a shift from resting toward effector states (Fig. 4I). By contrast, NR samples exhibited frequent interactions that remained predominantly in a resting morphologic state [43]. Collectively, these findings indicate that productive synaptic engagement was associated with morphologic T cell activation and plasma cell lysis, whereas non-productive engagement was not.

### TEC and TAL elicit target-specific effector dynamics confirmed by antigen-loss model

**Fig. 5:**
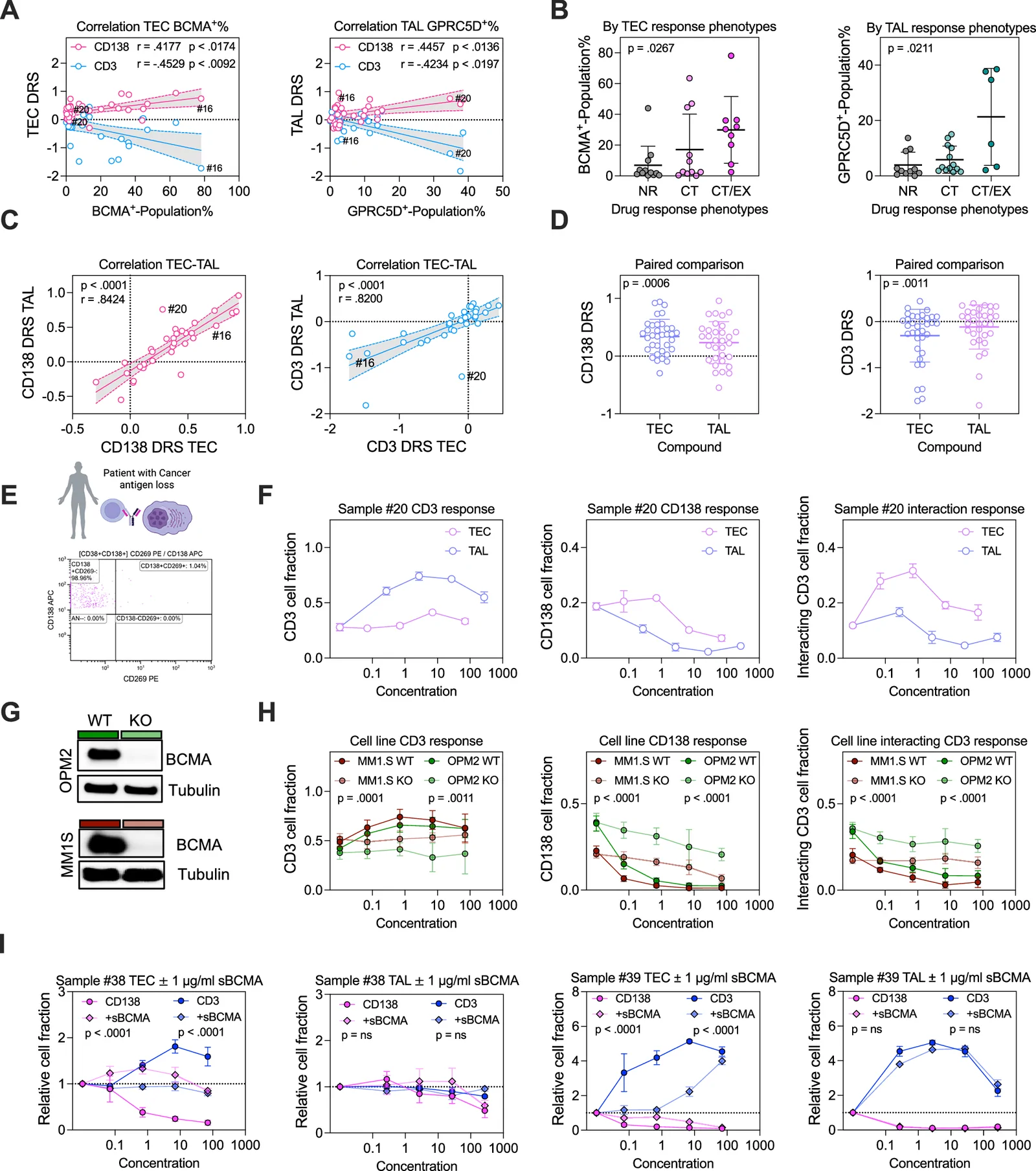
Target gene loss impairs plasma cell lysis and T cell expansion. **A** Correlations of CD138-DRS and CD3-DRS with the fraction of BCMA⁺ plasma cells (TEC; left) and GPRC5D⁺ plasma cells (TAL; right); *n* = 35 (TEC) and *n* = 32 (TAL) patient samples with flow cytometry data. **B** BCMA⁺ (TEC) and GPRC5D⁺ (TAL) plasma cell fractions by response phenotype; *n* = 35 (TEC) and *n* = 32 (TAL). **C** Correlation of CD138-DRS (left) and CD3-DRS (right) between TEC and TAL in matched samples. **D** Paired comparison of CD138-DRS and CD3-DRS between TEC and TAL in matched samples. **E** Example flow cytometry gating illustrating low BCMA expression in patient sample #20 (CD138/CD269 (BCMA)). **F** TEC and TAL dose–response curves for patient sample #20 showing control-normalized CD3⁺, CD138⁺, and interacting CD3⁺ fractions. **G** Immunoblot confirming CRISPR/Cas9-mediated BCMA knockout (KO) in OPM2 and MM1.S cell lines. **H** TEC dose–response curves in MM1.S and OPM2 WT versus BCMA KO co-cultured with PBMCs from three healthy donors (*n* = 3 donors), showing control-normalized CD3⁺, CD138⁺, and interacting CD3⁺ fractions. **I** TEC and TAL dose-response curves for patient samples #38 and #39 w/o presence of additional soluble BCMA (sBCMA) of 1 µg/ml. Data are presented as mean ± SD in (**B, D, F, H**). Line of best fit presented as mean + 95% CI in (**A, C**). Statistical analyses: Spearman’s rank correlation (**A, C**), Kruskal–Wallis test (**B**), Wilcoxon matched-pairs signed-rank test (**D**), two-way ANOVA with Tukey’s multiple-comparison test (**H, I**). Schematic representation in E created with BioRender.com. Abbreviations: WT wild type, KO knockout, TEC teclistamab, TAL talquetamab.

Given their comparable clinical efficacy but different target surface antigens, we next examined whether our assay could resolve TCE-specific differences ex vivo. Flow cytometric analysis of ex vivo*-treated* samples demonstrated that BCMA^+^ (TEC) and GPRC5D^+^ (TAL) plasma cell fractions correlated with increased plasma cell lysis and T cell expansion (Fig. 5A, B), indicating that antigen loss or low target expression was associated with attenuated ex vivo responses.

Despite high overall concordance between TEC and TAL across key readouts (Fig. 5C), TEC elicited stronger T cell expansion and more pronounced plasma cell lysis (Fig. 5D), whereas TAL yielded higher T cell–plasma cell interaction frequencies at 120 h (Supplementary Fig. 6A, B).

Sample #20 with minimal BCMA expression (Fig. 5E) after BCMA-CAR-T therapy showed partial plasma cell lysis to TEC but CT/EX response to TAL, consistent with antigen-specific resistance (Fig. 5F). Conversely, sample #16, which retained high BCMA expression (Supplementary Fig. 6C), responded with clonal expansion to both agents (Supplementary Fig. 6D).

To directly test whether antigen loss could account for discordant T cell expansion, we generated BCMA-deficient MM1.S and OPM2 myeloma cell lines using CRISPR/Cas9-mediated knockout (BCMA KO, Fig. 5G) and evaluated their responses to TEC in the image-based ex vivo assay. BCMA KO and BCMA wild-type (WT) cells were co-cultured with allogeneic PBMCs from three healthy donors and treated with increasing TEC concentrations for 72 h. As expected, WT co-cultures exhibited rapid CD138⁺ plasma cell lysis and CD3⁺ T cell expansion, whereas BCMA KO cells displayed attenuated CD138⁺ plasma cell lysis and absent CD3⁺ T cell expansion (Fig. 5H), recapitulating the cytotoxic phenotype observed in the BCMA-low patient sample. On the other hand, it has been shown that high soluble BCMA plasma levels hinder the efficiency of BCMA targeting TCEs [44]. To further analyze if high levels of sBCMA alter TEC or TAL efficiency ex vivo, we treated MM1S and OPM2 cell lines, as well as primary MM samples, in the presence of added sBCMA, decreasing cell killing efficiency of TEC but not TAL (Fig. 5I and Supplementary Fig. 6E).

### Ex vivo TCE-induced T cell expansion stratifies clinical response in a prospective patient cohort

**Fig. 6:**
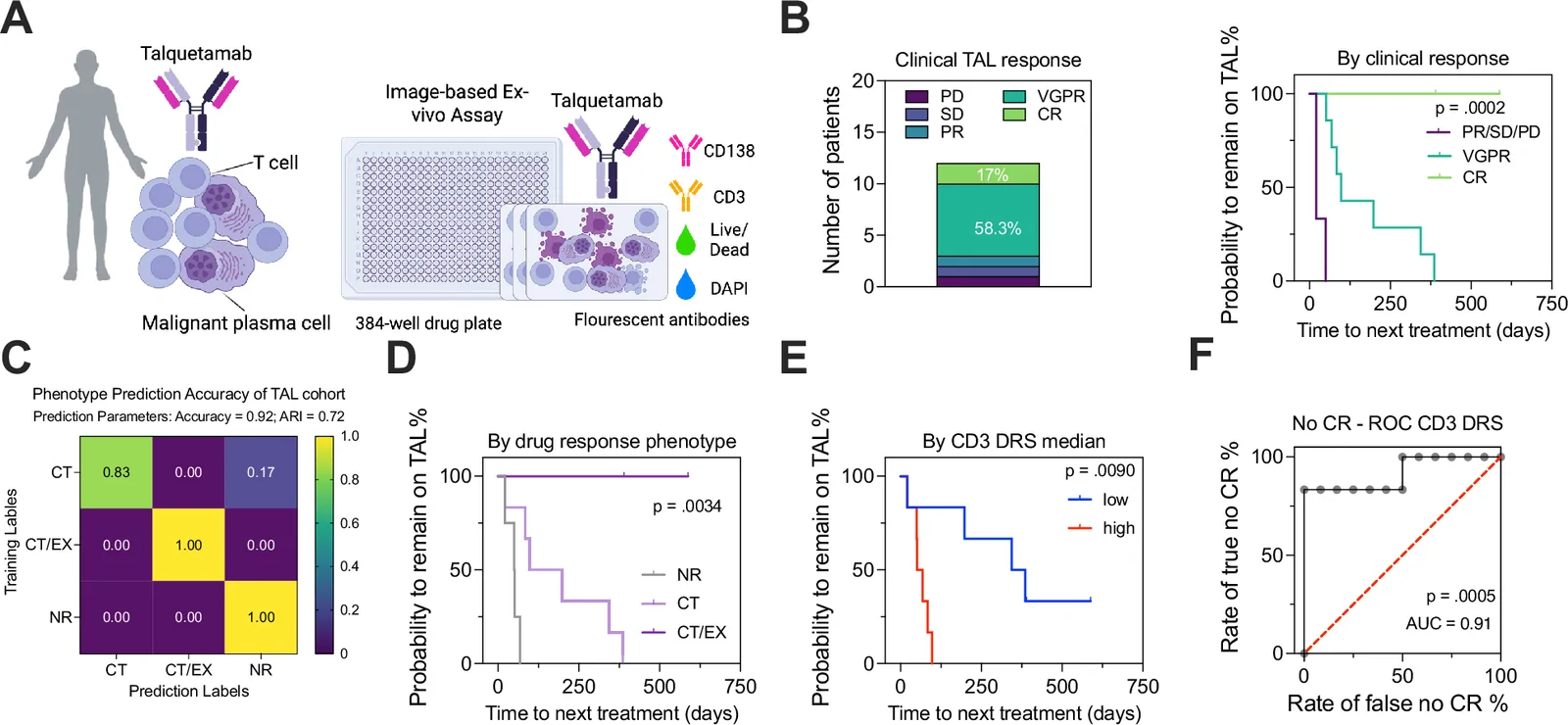
Ex vivo T cell expansion predicts clinical outcome in a talquetamab-treated cohort. **A** Overview of image-based ex vivo profiling performed on baseline MM BMMCs collected before TAL treatment. **B** Clinical best response categories during TAL therapy (PD, SD, PR, VGPR, CR) and Kaplan–Meier analysis of time on therapy by response group; *n* = 12 patients. **C** Confusion matrix showing phenotype assignment concordance between the training set (*n* = 32) and the TAL cohort (*n* = 12). **D** Kaplan–Meier analysis of time on TAL by ex vivo response phenotype (NR, CT, CT/EX); *n* = 12. **E** Kaplan–Meier analysis of time on TAL stratified by CD3-DRS (high vs low); *n* = 12. **F** Receiver operating characteristic (ROC) curve for CD3-DRS to discriminate CR (*n* = 2) from non-CR (*n* = 10). Censored observations are indicated on survival curves. Statistical analyses: log-rank test (**B, D, E**), Mann–Whitney U test (**F**). Schematic representation in A created with BioRender.com. Abbreviations: CT cytotoxic, CT/EX cytotoxic-expansive, NR non-responder, TAL talquetamab.

To assess the clinical relevance of the image-based ex vivo assay, we prospectively analyzed baseline BMMCs from 12 patients (Supplementary Table 1) who subsequently received TAL (Fig. 6A). The TAL cohort was heavily pretreated, including patients with penta-drug refractoriness, prior CAR-T exposure, and extramedullary disease. None of these baseline disease characteristics stratified clinical outcomes (Supplementary Table 2).

Time to next treatment was used as the clinical outcome parameter [25]. Median time to next treatment was 98 days (range: 21–397 days) (Supplementary Fig. 7A). Among the 12 patients, two achieved complete remissions (CR), seven very good partial responses (VGPR), one partial response (PR), one progressive disease (PD) and one patient had stable disease at assessment but died due to septic disease (Fig. 6B). Depth of clinical response correlated strongly with time on TAL (log-rank *p* = 0.0002), supporting the clinical representativeness of the cohort (Fig. 6B).

To test our assay’s predictive capacity, we stratified by ex vivo drug response phenotype. Re-clustering of the TAL cohort reproduced labels with 92% accuracy (Fig. 6C). Stratification by ex vivo drug response phenotype demonstrated clear separation in time on TAL across NR (*n* = 4), CT (*n* = 5), and CT/EX (*n* = 3) groups (log-rank *p* = 0.0034) (Fig. 6D and Supplementary Fig. 7C). This association remained significant after exclusion of patients receiving TAL as bridging therapy prior to CAR-T (log-rank *p* = 0.0294) (Supplementary Fig. 7D). Because the ex vivo drug response phenotypes integrate both T cell expansion and plasma cell lysis, we next tested these parameters individually. Higher ex vivo T cell expansion (negative CD3 DRS) was strongly associated with prolonged time to next treatment (log-rank *p* = 0.009) (Fig. 6E and Supplementary Fig. 7E), whereas plasma cell lysis (positive CD138 DRS) showed a similar trend without reaching significance (Supplementary Fig. 7F, G).

Receiver operating characteristic analysis demonstrated that both CD138-DRS (AUC = 0.87, *p* = 0.0018) (Supplementary Fig. 7H) and CD3-DRS (AUC = 0.91, *p* = 0.0005) (Fig. 6F) discriminated patients achieving CR from those with lower response depth. Finally, the morphologic T cell activation parameter rTC-iAUC_norm_ also discriminated CR from non-CR cases (AUC = 0.95, *p* = 0.0001) (Supplementary Fig. 7I).

Taken together, these findings reveal that ex vivo response phenotypes stratified clinical response and time on therapy in TAL-treated patients. Among the assessed functional parameters, T cell expansion showed the strongest association with clinical benefit.

## Discussion

In this study, we show that image-based ex vivo profiling can resolve TCE pharmacodynamics in MM BMMCs and stratify clinical outcomes. By integrating plasma cell lysis, T cell expansion, single-cell morphology, and synaptic engagement, we identified three ex vivo drug response phenotypes and delineated a morphologic T cell activation trajectory from resting to effector TCM that correlates with ex vivo efficacy.

A central insight is that morphologic activation encodes functional T cell fitness in BMMCs from MM patients. Along a morphologic continuum from resting to polarized and effector TCM, we observed cytoplasmic remodeling, nuclear enlargement, and CD3 redistribution in association with plasma cell lysis and T cell expansion. In practical terms, T cell morphology may serve as a compact surrogate for T cell signaling and cytolytic competence within patient-derived BMMCs. Prior imaging and deep-learning studies have linked T cell shape and texture to signaling and proliferative potential, and our results extend these observations to primary MM BMMCs under TCE stimulation by anchoring morphology to functional killing [26–28, 31, 36, 37, 39].

Equally important, our data indicate that engagement quality, rather than contact quantity, distinguishes response. TCE-responding samples accumulated composition-corrected interaction enrichment over time, yet displayed lower fractions of interacting T cells within the T cell population at later time points. This pattern is consistent with productive serial engagement, timely synapse resolution, target-cell clearance, and subsequent T cell expansion [34, 42, 43]. By contrast, non-responding samples displayed frequent contacts in which T cells remained predominantly morphologically resting, suggesting abortive synapses that fail to propagate effective activation. Integrating TCM annotations with spatial interaction metrics provided a unified and mechanistically interpretable view of the ex vivo response heterogeneity across patient samples.

Beyond ex vivo drug response and morphologic T cell activation, our assay provided functional evidence for antigen-specific resistance. This was exemplified by a sample with reduced BCMA expression that showed limited response to TEC but preserved sensitivity to TAL, consistent with target dependence. We further validated this concept using CRISPR/Cas9-mediated BCMA knockout models, demonstrating that BCMA loss can attenuate plasma cell lysis and abrogate clonal T cell expansion despite otherwise competent allogeneic effector cells [13, 14]. Together, these data support antigen availability as a key determinant of primary functional response in this setting.

Translationally, our findings align with prior functional precision medicine efforts, such as the EXALT-I trial, and extend them to TCEs [20, 21]. In our prospectively analyzed cohort, ex vivo T cell expansion stratified time on therapy, underscoring potential clinical utility. In practice, image-based profiling could (i) triage patients toward the TCE most likely to succeed in their cellular ecosystem, (ii) identify cases where insufficient functional T cell capacity may warrant priming or co-stimulatory strategies, and (iii) enable longitudinal pharmacodynamic monitoring to detect emerging resistance. Moving forward, future studies should evaluate this approach prospectively in larger, multi-center cohorts and across different TCE regimens and disease stages and should clarify how ex vivo readouts perform alongside established clinical and molecular biomarkers.

In summary, image-based ex vivo profiling condenses the complexity of TCE pharmacodynamics into reproducible, mechanistically grounded features with clear translational relevance. By capturing both immune effector dynamics and plasma cell fate at single-cell resolution, this approach establishes a framework for functional patient stratification.

Beyond proof-of-concept, these findings provide a foundation for the development of predictive, functionally validated biomarkers to guide the personalized deployment of TCE therapy in MM, thereby bridging mechanistic immunology and precision oncology. Furthermore, to facilitate the translation of this assay into clinical practice, ongoing efforts include its expansion to encompass additional drug combinations, as well as the implementation of a clinical trial to assess its utility for identifying optimal combination strategies for individual patients.

## Supplementary information

Supplementary Data and Figures

## Data Availability

The datasets generated and analyzed during the current study are available from the corresponding author upon reasonable request.
