# Supplementary material for: Functional immune profiling translates T cell dynamics into predictive biomarkers for myeloma immunotherapy
Source: Leukemia. 2026 Jul 16;40(9):1997–2007. doi: 10.1038/s41375-026-03047-5 (PMC13506338; doi:10.1038/s41375-026-03047-5)
Supplement: Supplementary file 1 [file 41375_2026_3047_MOESM1_ESM.pdf]

Supplementary Data:

Supplementary Table 1

ID	age	disease stage	ISS	EM	cytogenetics	IMiD	PI	CD38mAB	drug class refractory	tal response	time to best response
12	62	r/r	1	no	highrisk	refractory	exposed	refractory	2	VGPR	86
13	63	r/r	3	yes	unknown	refractory	refractory	refractory	3	SD	32
14	73	r/r	3	no	highrisk	refractory	refractory	refractory	5	VGPR	66
15	61	r/r	1	yes	standard	refractory	exposed	refractory	2	sCR	59
16	64	r/r	2	no	standard	refractory	refractory	refractory	5	VGPR	60
17	72	r/r	2	no	unknown	refractory	exposed	refractory	2	VGPR	76
18	74	r/r	3	no	unknown	refractory	refractory	refractory	3	VGPR	31
19	75	r/r	3	yes	standard	refractory	refractory	refractory	3	VGPR	63
20	61	r/r	2	no	standard	refractory	refractory	refractory	5	sCR	37
21	58	r/r	1	no	standard	refractory	refractory	refractory	3	PR	48
23	70	r/r	3	yes	highrisk	refractory	refractory	refractory	5	PD	/
35	67	r/r	2	0	standard	refractory	refractory	refractory	5	VGPR	84

sTab1: Clinical characteristics of talquetamab treated cohort (EM= extra medullary disease, DCR= drug class resistance; IMID= immunomodulatory drug PI= proteasome inhibitor; VGPR= very good partial remission, SD= stable disease, (s)CR= stringent complete remission, PR= partial remission, PD= progressive disease)

Supplementary Figure 1

(**A**) Viable cell fractions after 24, 72 and 120h; n = 10. (**B**) teclistamab response curves of control normalized CD3 (left) and CD138 (right) cell fractions after 24, 72 and 120 h; n = 10. (**C**) talquetamab response curves of control normalized CD3 (left) and CD138 (right) cell fractions after 24, 72 and 120 h; n = 10. (**D**) Distribution of plasma cell percentage across all samples. (**E**) Response to TEC and TAL by plasma cell percentage. (**F**) Correlation of DRS score of CD3 and CD138 to TEC and TAL depending plasma cell percentage. (**G**) Drug synergy map of patient sample #36 and #37 of combining TEC and TAL. (**H-K**) Comparison of CD138 and CD3 DRS to TEC and TAL depending on triple or penta drug class refractoriness. Data are presented as mean in A and as mean ± SEM in B and C. Statistical significance was determined by Two-way-ANOVA with Tukey's multiple-comparison test **A, B** and **C**. P value of column factor (time) is indicated in plots. **G** analysis and illustration by synergyfinder.org (TEC= teclistamab, TAL=talquetamab)

Supplementary Figure 2

(**A**) Example of Cell Painting analysis workflow in Harmony 5.1: Pseudocoloured image of T cells (CD3+,yellow). Nucleus appears blue (DAPI) and Live/Dead green (left). CD3 channel on greyscale (center). Segmentations of CD3+cells (right). Identical linear contrast was applied across all images. Scale bar, 10 µm.(**B**) Cell region segmentations. Nucleus (green), Nuclear Ring (red), Cytoplasm (blue), Membrane (pink) and Cell (yellow). Scale bar, 10 µm.

Supplementary Figure 3:

(**A**) Comparison of resting T cells (rTC), early activated T cells (eaTC), polarized T cells (polTC) and effector T cells (eTC) iAUCnormbetween drug response phenotypes for teclistamab; n= 36. (**B**) Comparison of resting T cells (rTC), early activated T cells (eaTC), polarized T cells (polTC) and effector T cells (eTC) iAUCnorm between drug response phenotypes for talquetamab; n= 33. In scatter plots each dot represents a single patient sample. Data are presented as mean stacked bar plot in **A** and **B**. Statistical significance was determined by Kruskal Wallis test in **A** and **B.** (TEC = teclistamab, TAL=talquetamab, no response = NR, cytotoxic only response= CT, cytotoxic-expansion response = CT/EX)

Supplementary Figure 4:

(**A**) Example of Cell-Cell-Interaction analysis workflow in Harmony 5.1: Pseudo-coloured image of T cells (CD3+, yellow) and plasma cells (CD138+, magenta). Nucleus appears blue (DAPI) and Live/Dead green (left). Segmentations of CD3+ and CD138+ cells. CD3+ membranes appear purple, CD138+membranes green (center). CD3 Channel on greyscale. Interacting CD3+ cells are highlighted in green, non-interacting CD3+ cells in red. Identical linear contrast was applied across all images. Scale bar, 10 µm. (**B**) Log2 rel. Interaction Enrichment curve induced by teclistamab (left) or talquetamab (right) after 24, 72 and 120 h; n = 10. (**C**) Log2 rel. Interaction

Enrichment response curve induced by teclistamab (left) and talquetamab (right) after 120 h; n = 36 (teclistamab), n= 33 (talquetamab). (**D**) Correlation of CD3 DRS and CD138 DRS log2 rel. Interaction Enrichment for teclistamab and talquetamab; n = 69. (**E**) Quantified morphologic shift by interacting morphotype cell fraction induced by teclistamab and talquetamab; n = 36 (teclistamab), n = 33 (talquetamab). (**F**) UMAP of single interacting T cell morphology coulored by morphotype cluster; n = 21734 cells (teclistamab), n = 20712 cells (talquetamab). (**G**) Comparison of eaTC and polTC iAUCnorm between drug response phenotypes for teclistamab; n= 36. (**H**) Correlations of iAUCnorm with CD3 and CD138 DRS of both BiTEs for interacting eaTC and polTC; n = 36 (teclistamab), n = 33 (talquetamab). (**I**) Comparison of interacting eaTC and polTC iAUCnorm between drug response phenotypes for talquetamab; n= 33. In scatter plots each dot represents a single patient sample. Data are presented as mean ± SD in **C, G** and **I**, as mean ± SEM in **B**. Line of best fit presented as mean + 95% CI in **D** and **H**. Statistical significance was determined by Two-way-ANOVA with Tukey's multiple comparison test in **B**, Spearman Correlation in **D** and **H** and Kruskal Wallis test in **G** and **I.** (TEC = teclistamab, TAL=talquetamab, no response = NR, cytotoxic only response= CT, cytotoxic-expansion response = CT/EX)

Supplementary Figure 5:

(**A**) Gating strategy to determine the fraction of BCMA and GPRC5D positive plasma cells from frozen BMMC (BMMCs = bone marrow mononuclear cells)

(**A**) Correlations of peTC iAUCnorm between teclistamab and talquetamab; n = 33. (**B**) Paired comparison of peTC iAUCnorm norm between teclistamab and talquetamab; n = 33 (**C**) Schematic representation and diagnostic flow dot plot of CD138-APC-CD269-PE indicating BCMA expression in patient sample #16. (**D**) teclistamab and talquetamab response curves from patient sample #16 of CD3, CD138 and CD3int cell fractions. Schematic representation created with biorender.com. (TEC = teclistamab, TAL=talquetamab, BMMCs = bone marrow mononuclear cells)

Supplementary Figure 6:

(**A**) Correlations of peTC iAUCnorm between teclistamab and talquetamab; n = 33. (**B**) Paired comparison of peTC iAUCnorm norm between teclistamab and talquetamab; n = 33 (**C**) Schematic representation and diagnostic flow dot plot of CD138-APC-CD269-PE indicating BCMA expression in patient sample #16. (**D**) teclistamab and talquetamab response curves from patient sample #16 of CD3, CD138 and CD3int cell fractions. (**E**) TEC dose–response curves in MM1.S and OPM2 w/o added soluble BCMA co-cultured with PBMCs from three healthy donors (n = 3 donors), and negative synergy map between TEC and soluble BCMA. (**F**) Circos plot of patient sample #40 providing genomic loss of GPRC5D and TEC and TAL dose–response showing control-normalized $CD3^+$, $CD138^+$, and interacting $CD3^+$ fractions. Schematic representation created with biorender.com. (TEC = teclistamab, TAL=talquetamab, BMMCs = bone marrow mononuclear cells)

Supplementary Figure 7:

(**A**) Survival curve indicating probability to remain on TAL treatment. (**B**) Patients grouped by drug response phenotype NR, CT and CT/EX; n = 12. (**C**) Survival curve indicating probability to remain on treatment between NR and CT & CT/EX phenotypes; n = 12. (**D**) Survival curve indicating probability to remain on treatment between NR and CT and CT/EX phenotypes with CAR-T bridging patients excluded; n = 8. (**E**) Survival curve indicating probability to remain on treatment between CD3 DRS high and low with CAR-T bridging patients excluded; n = 8. (**F**) Patients grouped by ranked CD138 DRS into high and low; n = 12. (**G**) Survival curve indicating probability to remain on treatment between high and low CD138 DRS group. Survival curve indicating probability to remain on treatment between

CD138 DRS high and low with CAR-T bridging patients excluded; n = 8. (**H, I**) Receiver-operating characteristic (ROC) curves showing the ability CD138-DRS (**H**) and rTC iAUCnorm (**I**). (**J**) Scatter Plot comparing rTC iAUCnorm between patients achieving a CR (n = 2) and SD/PD/PR/ VGPR (n =10). In scatter plots each dot represents a single patient sample. Data are presented as mean ± SD in **J**. Censored data points are highlighted as dots on survival curves in **A**, **C, D, E, F, G**. Statistical significance was determined by Log rank test in **A**, **C, D, E, F, G** and Man Whitney U test in **H**, **I**, **J. (**TAL= talquetamab**)**

Supplementary Methods:

Lentiviral transduction and CRISPR/Cas9-mediated gene deletion

OPM2 and MM1.S cells were transduced with Cas9 (pLKO5d.SSF.SpCas9.P2a.BSD) and selected with blasticidin. SgRNAs targeting TNFRSF17 (BCMA) were cloned into lentiGuide-Puro. Lentiviral particles were generated in HEK293T cells using psPAX2 and pMD2.G packaging plasmids. After transduction, cells were selected with puromycin (2 μg/mL). Knockout efficiency was confirmed by Western blot.

Western Blot

Cells were lysed in RIPA buffer, and protein concentrations quantified by Bradford assay. Proteins (15 μg/lane) were separated by SDS-PAGE, transferred to PVDF membranes, and probed with anti-TNFRSF17 (Proteintech), anti-Tubulin (Sigma), and appropriate HRP-conjugated secondaries. Immunoreactive proteins were visualized via ECL chemiluminescent detection (Advansta, San Jose, USA).

Flow Cytometry

Flow cytometry staining was performed according to standard protocols. Briefly, frozen BMMC were thawed, washed and stained with Zombie-NIR, CD229-BV400, CD319-BV421, GPRC5D-AF700 and CD269-Dazzle594 (BCMA) antibodies at 37 °C for 30 min. Data were collected on a Cytek Aurora (Cytek Biosciences, USA) and analyzed with FlowJo software (FlowJo, LLC; V. 10.10).

Find variable morphology features

To find variable features between morphology clusters, morphological features were z-scaled. For each feature we tested differences across morphology clusters using a Kruskal–Wallis test. P-values were adjusted with Benjamini–Hochberg (FDR). Features with FDR < 0.05 were deemed cluster-associated and ranked by the Kruskal–Wallis H statistic (effect size). For the heatmaps, we then selected the top 5 features per cluster based on the largest absolute median shift (cluster median minus pooled median), retaining the direction (higher/lower in that cluster) and avoiding duplicates, yielding 20 features total for four clusters. Cluster summaries shown in heatmaps are cluster means (on the z-scale).

Supplementary Table 2

Characteristic	n (%) or median (range) or p-value (Log-rank)
**Age at sample**	67.9 (57 - 75)
**Sex**	p = 0.6718
female	5 (41.66%)
male	7 (58.83%)
**ISS stage III**	p = 0.8476
ISS I	3 (25.00%)
ISS II	4 (33.33%)
ISS III	5 (41.66%)
**Extramedullary disease (EMD)**	p = 0.9835
No EMD	8 (66.66%)
EMD	4 (33.33%)
**Last Therapy prior sample**	p = 0.6678
Elo/Pom/Dex	5 (41.66%)
Dara/Carf/Dex	1 (8.33%)
Dara/Pom/Dex	2 (16.66%)
Elotuzumab	1 (8.33%)
Ixa/Len/Dex	1 (8.33%)
Cilta-cel	2 (16.66%)
**Drug refractory status**	p = 0.4328
2	3 (25.00%)
3	4 (33.33%)
5	5 (41.66%)
**Prior CAR-T therapy**	p = 0.1422
No prior CAR-T	8 (66.66%)
Prior CAR-T	4 (33.33%)

sTab2: baseline disease characteristics stratified by outcome

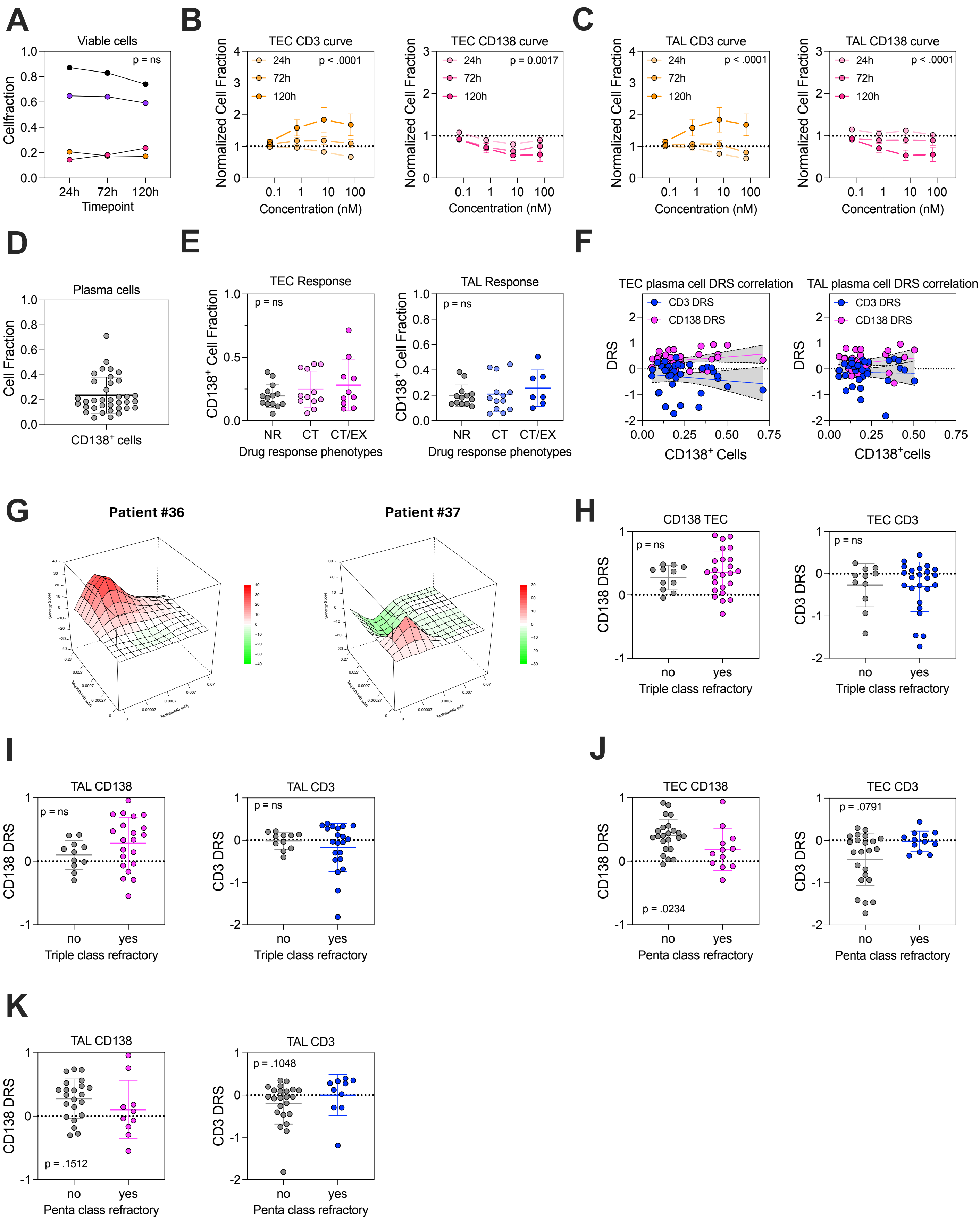

Supplementary Figure 1

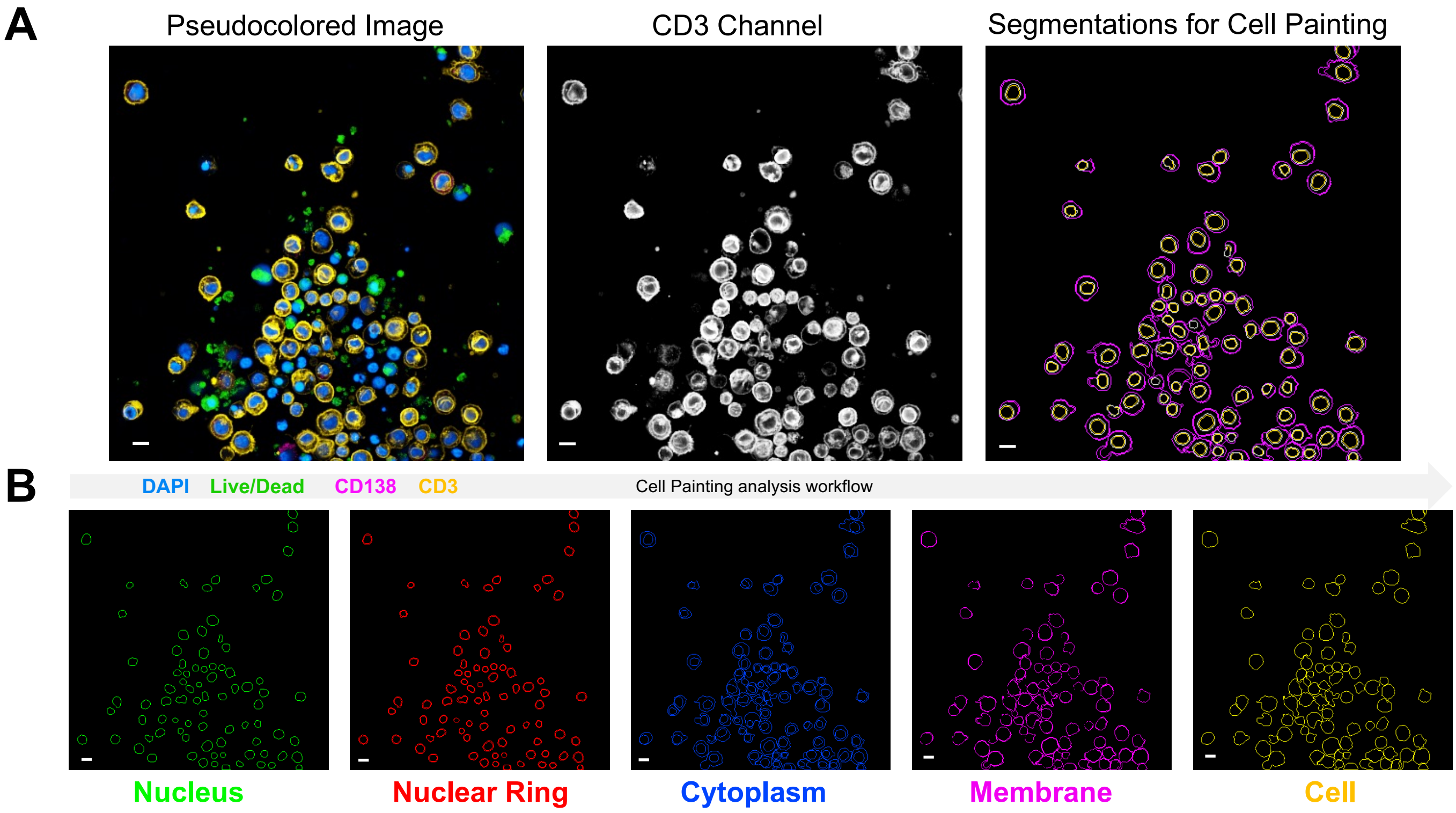

Supplementary Figure 2

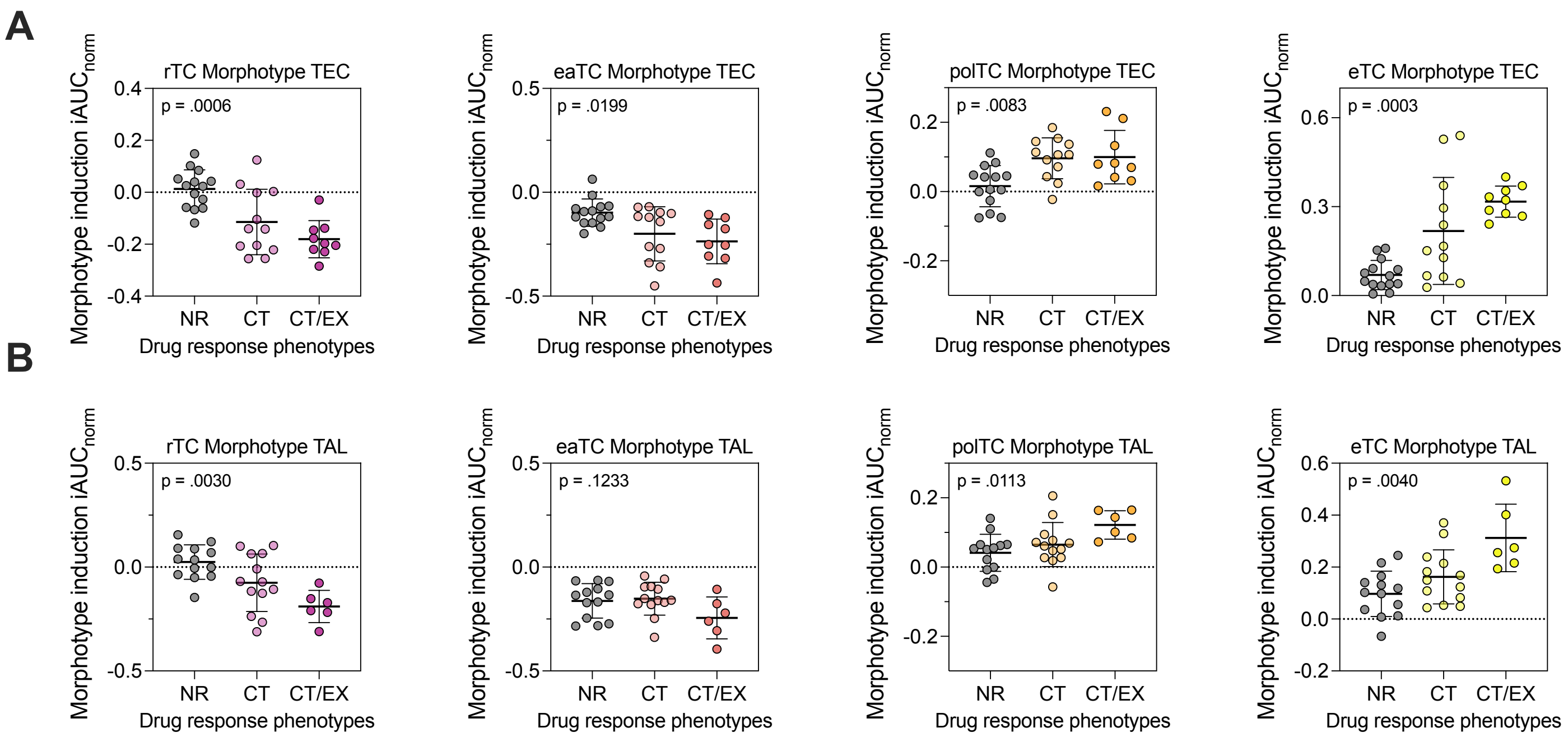

Supplementary Figure 3

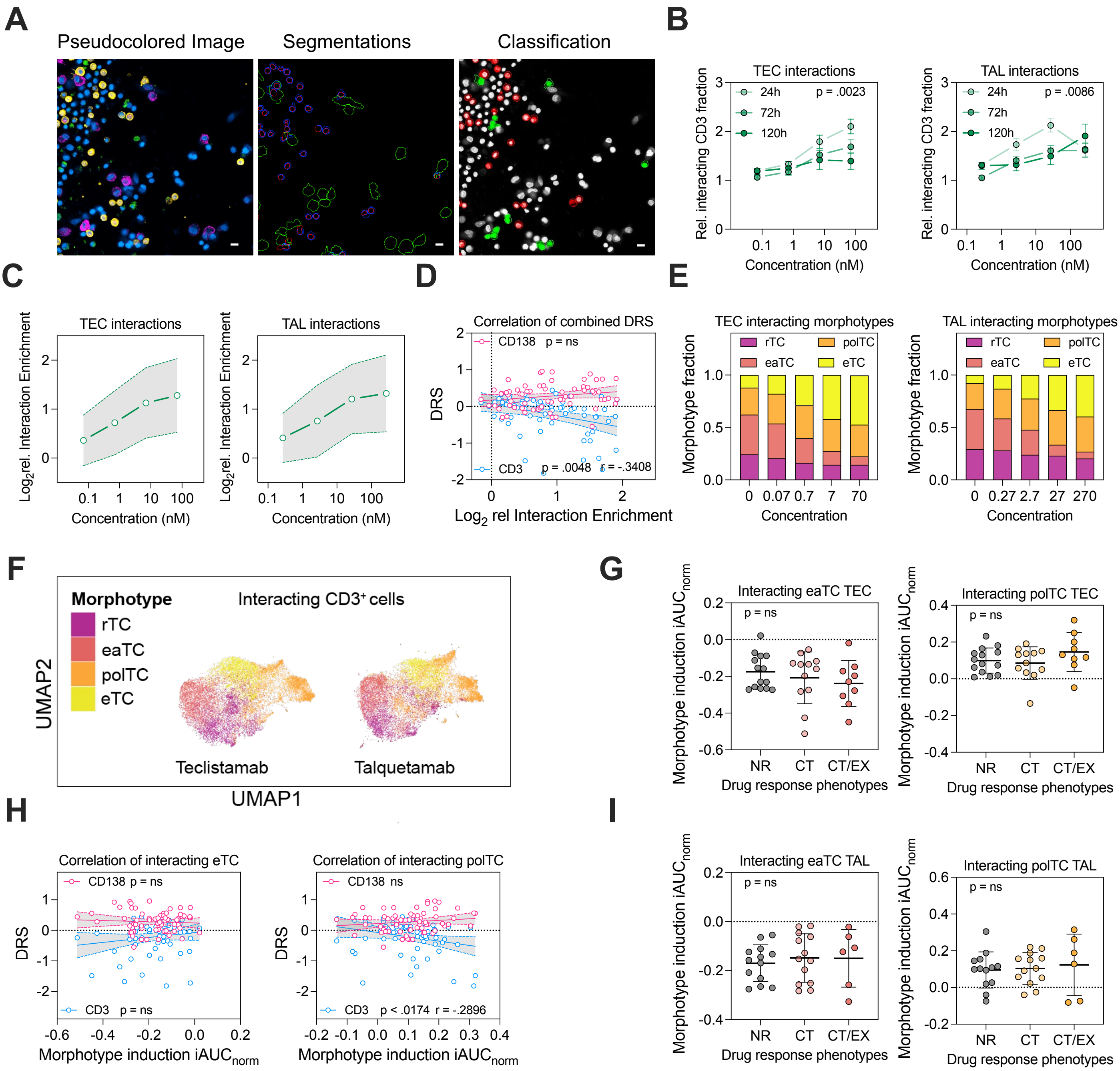

Supplementary Figure 4

A

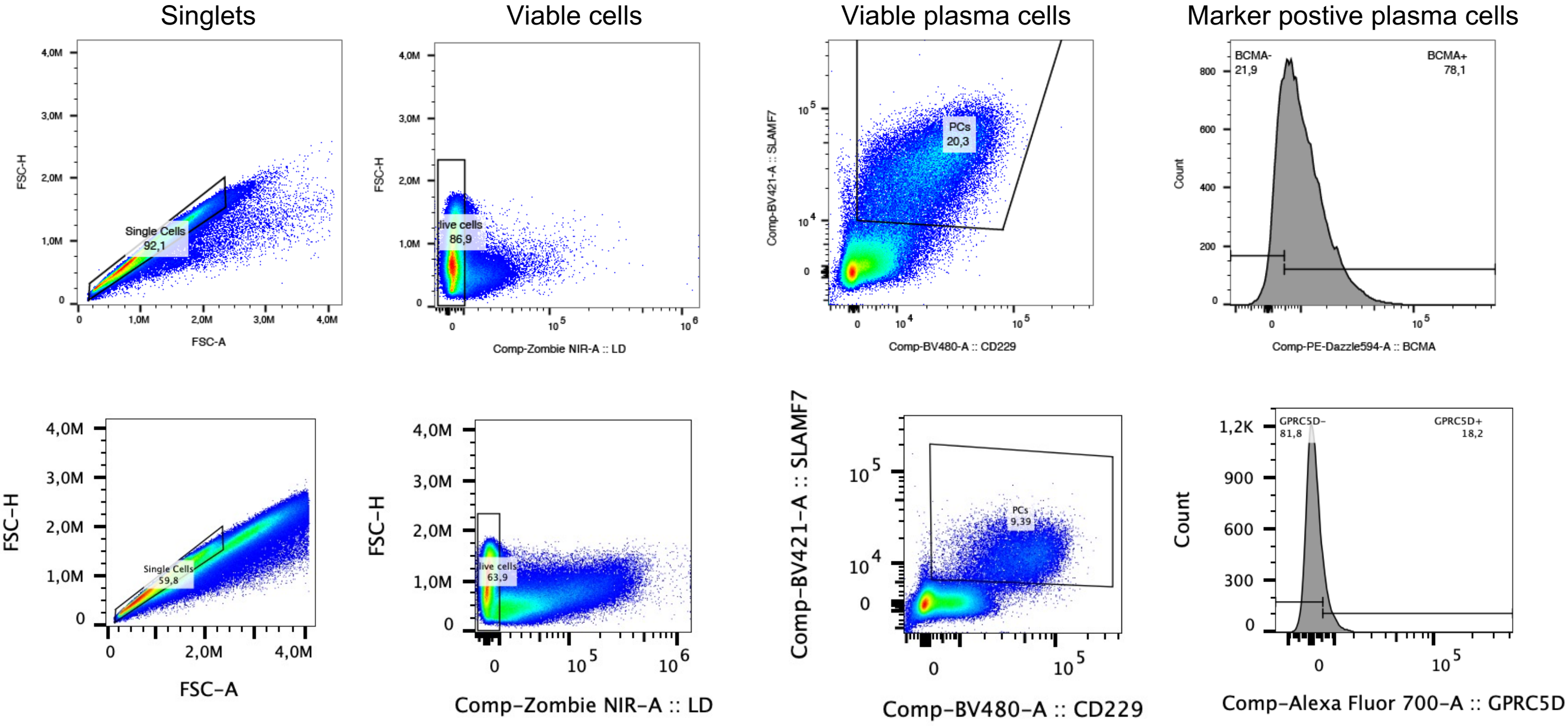

Supplementary Figure 5

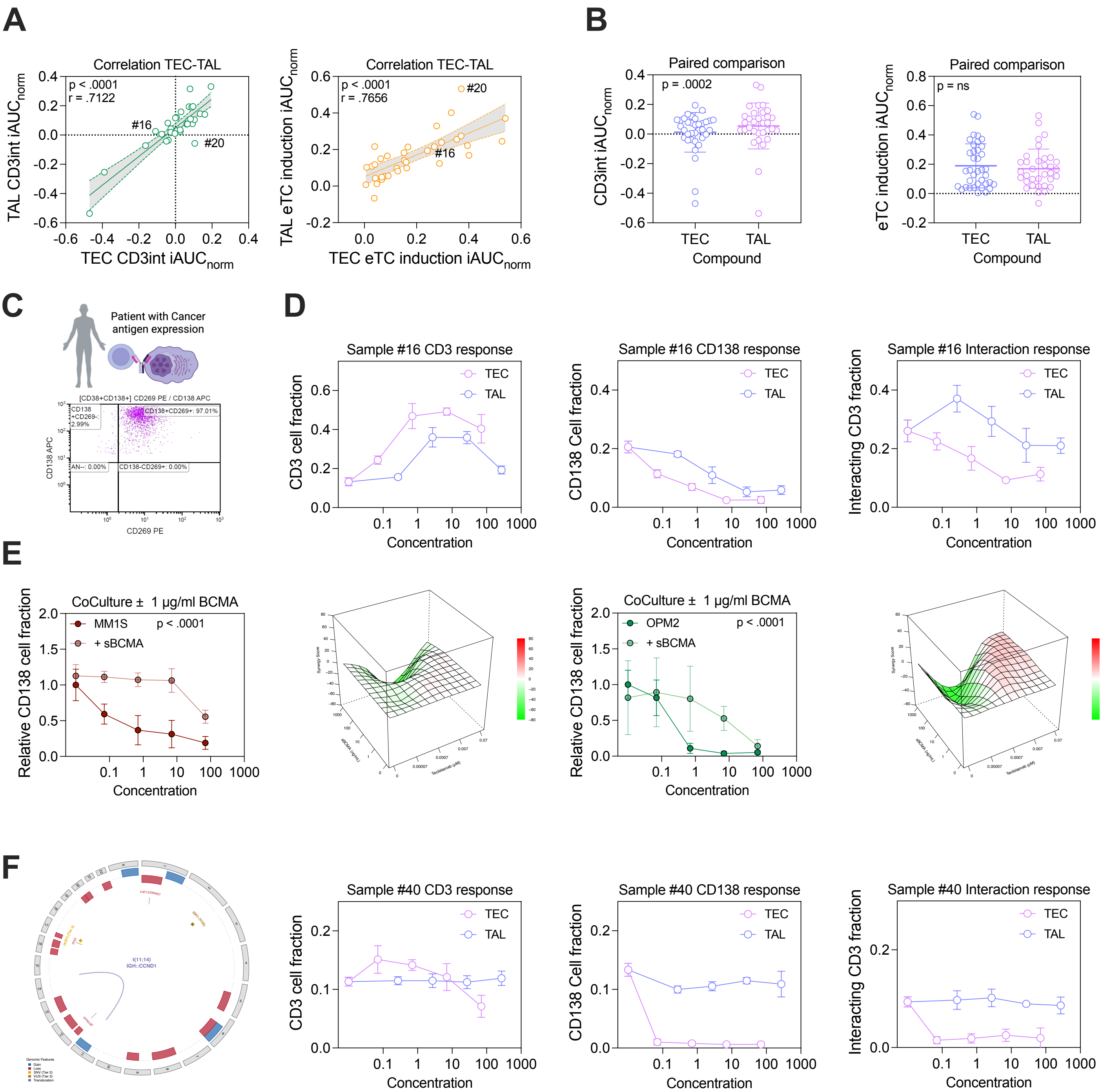

Supplementary Figure 6

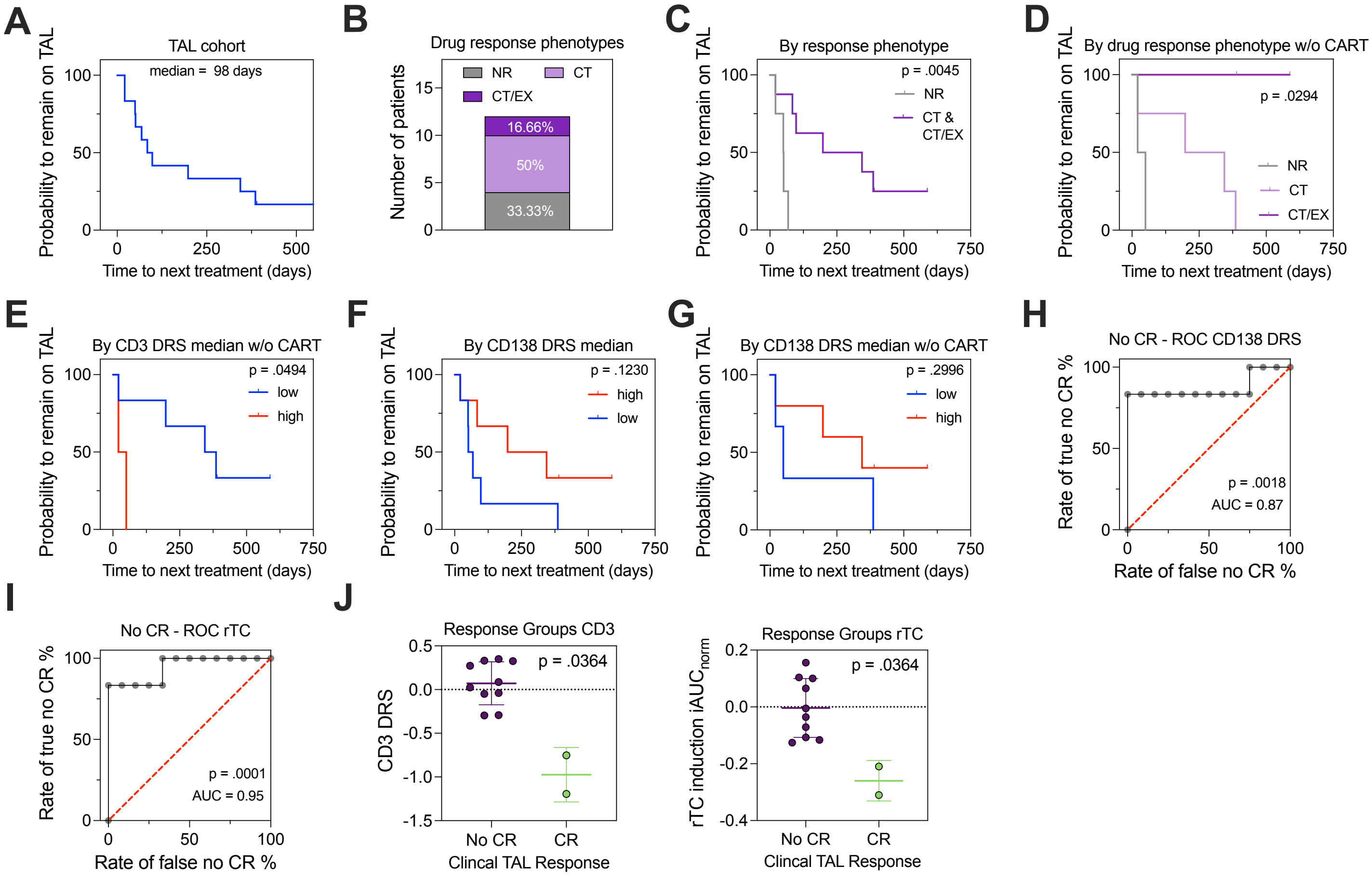

Supplementary Figure 7
